# Safety of thoracic radiotherapy after PD‐(L)1 inhibitor treatment in patients with lung cancer

**DOI:** 10.1002/cam4.4363

**Published:** 2021-10-19

**Authors:** Yu Chen, Xinchao Liu, Zhaoqin Huang, Kaikai Zhao, Yao Wang, Fei Ren, Jinming Yu, Xiangjiao Meng

**Affiliations:** ^1^ Cheeloo College of Medicine Shandong University Jinan Shandong China; ^2^ Department of Radiation Oncology Shandong Cancer Hospital and Institute Shandong First Medical University and Shandong Academy of Medical Sciences Jinan Shandong China; ^3^ Department of Radiology Shandong Provincial Hospital Affiliated to Shandong First Medical University Jinan Shandong China; ^4^ Department of Radiation Oncology Yantai Affiliated Hospital of Binzhou Medical University Yantai Shandong China

**Keywords:** lung cancer, PD‐(L)1 inhibitor, pneumonitis, safety, thoracic radiotherapy (TRT)

## Abstract

**Background:**

The safety of thoracic radiotherapy (TRT) after programmed death 1/programmed death ligand 1 (PD‐(L)1) inhibitor treatment in patients with lung cancer was scarcely reported. This retrospective study was conducted to evaluate the incidence, severity, and risk factors of symptomatic treatment‐related pneumonitis in patients with lung cancer who received this sequential combination.

**Methods:**

We conducted a retrospective study of a cohort of patients with lung cancer who received TRT after at least two cycles of PD‐(L)1 inhibitor treatment between January 2018 and August 2020. Treatment‐related pneumonitis was evaluated and analyzed to illustrate the safety profile of this sequential combination. Potential risk factors were explored by univariate and multivariate logistic regression analyses.

**Results:**

Among the 828 patients with prior PD‐(L)1 inhibitor treatment, 96 patients receiving subsequent TRT were included in the analysis. Of these, 49 patients (51%) received radical TRT while 47 patients (49%) received palliative TRT. The median total dose was 52 Gy (IQR 50–60 Gy). The median time from the initiation of PD‐(L)1 inhibitor treatment to TRT was 4.8 months (1.6–14.1 months) with most of the patients (74%) administering no less than four cycles of PD‐(L)1 inhibitor. During follow‐up, 47 patients (48.96%) developed symptomatic treatment‐related pneumonitis (grade 2 *n* = 28, grade ≥3 *n* = 19) while six patients (6.25%) suffered from fatal toxicity. The median time of pneumonitis onset after completion of TRT was 35 days (0–177 days) with six patients developing during TRT. Pulmonary emphysema and lung V20 were demonstrated to be independent risk factors of symptomatic pneumonitis (OR: 5.67, 95% CI: 1.66–19.37, *p* = 0.006; OR: 3.49, 95% CI: 1.41–8.66, *p* = 0.007, respectively).

**Conclusion:**

TRT after PD‐(L)1 inhibitor treatment resulted in significantly increased incidence and severity of treatment‐related pneumonitis in patients with lung cancer. Intensive attention should be emphasized to the safety of this sequential combination in clinical practice.

## Graphical abstract

The safety of thoracic radiotherapy (TRT) after PD‐(L)1 inhibitor treatment in patients with lung cancer was scarcely reported. We conducted a retrospective study of 96 patients with lung cancer who received TRT after multi‐cycle of PD‐(L)1 inhibitor treatment. A substantially increased incidence and severity of symptomatic pneumonitis was observed. It matters to weigh the risks against the benefits of this sequential regimen.

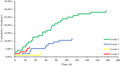

## 1 INTRODUCTION

Immune checkpoint inhibitors targeting programmed cell death (ligand) 1 (PD‐(L)1) and others have revolutionized the treatment paradigm in various cancers especially in lung cancer.^1^, ^2^, ^3^, ^4^ Though with potent clinical efficacy, a large proportion of patients suffer from disease progression after several cycles of PD‐(L)1 inhibitor treatment. Specifically, for patients with local progression or oligoprogression in the chest, subsequent TRT might be an appropriate option for salvage therapy. Notably, based on the evidence of IMpower 133 trial, CASPIAN trial, and CREST trial,^4^, ^5^, ^6^ it becomes a hot topic whether TRT should be considered to conduct in patients with small cell lung cancer (SCLC) who respond well to prior PD‐(L)1 inhibitor treatment for consolidation therapy.

Based on the fact that PD‐(L)1 inhibitor and TRT both can damage the lung,^7^, ^8^ there is a theoretical concern that PD‐(L)1 inhibitor combined with TRT irrespective of concurrent or sequential might result in enhanced pulmonary toxicities. Nowadays, available data, though showed a generally acceptable safety profile, mainly focused on the pulmonary toxicities of concurrent combination of PD‐(L)1 inhibitor with TRT or sequential combination of PD‐(L)1 inhibitor with a prior history of TRT.^2^, ^9^, ^10^, ^11^, ^12^ Whether regimen of TRT after multicycles of PD‐(L)1 inhibitor‐ induced tolerable pulmonary toxicities was scarcely reported. With the increasing use of PD‐(L)1 inhibitor particularly in first‐line treatment, it is conceivable that a growing number of patients with lung cancer will be exposed to subsequent TRT. Considering the unique immunomodulatory effects and complex interactions of PD‐(L)1 inhibitor and TRT,^13^ the sequence of the combination regimen might be associated with different efficacy and safety profile.

Herein, we aim to evaluate and characterize the incidence and severity of treatment‐related pneumonitis in a cohort of patients treated with TRT subsequently after multicycles of PD‐(L)1 inhibitor. Risk factors are explored to identify the patients who are prone to develop symptomatic pneumonitis.

## 2 PATIENTS AND METHODS

### 2.1 Patient population

We retrospectively identified a cohort of patients with lung cancer who underwent TRT after at least two cycles of PD‐(L)1 inhibitor treatment from January 2018 to August 2020 at Shandong Cancer Hospital and Institute. Patients irrespective of responding well or poorly to PD‐(L)1 inhibitor treatment were included if they received subsequent TRT targeting lung parenchyma, draining lymph nodes, or both. For patients without treatment‐related pneumonitis, the clinical follow‐up was required to be no less than 6 months after completion of TRT. Patients who had already developed symptomatic treatment‐related pneumonitis before TRT, received thoracic reirradiation, or lack of consecutive information for the evaluation of pneumonitis were excluded. Baseline computed tomography (CT) images before TRT were required to exclude patients with preexisted pneumonitis. Detail data on baseline characteristics, dosimetric parameters of TRT, radiographic findings, and other results were extracted from electronic medical records. This study was approved by the institutional review board (IRB) of Shandong Cancer Hospital and Institute.

### 2.2 Treatment regimen

All of the enrolled patients received PD‐(L)1 inhibitor as monotherapy or in combination with chemotherapy regardless of treatment lines. The specific PD‐(L)1 inhibitors were comprised of pembrolizumab, nivolumab, durvalumab, and others. The specific medication regimen of PD‐(L)1 inhibitor for patients was administered according to the Chinese Society of Clinical Oncology (CSCO) guidelines or National Comprehensive Cancer Network (NCCN) guidelines. Patients were treated with three‐dimensional conformal RT (3D‐CRT) or intensity‐modulated RT (IMRT). The dosimetric parameters were extracted from the treatment planning system (Eclipse system, Varian Medical Systems, Version 13.5.35). Target volumes were delineated followed the guidelines of Radiotherapy and Oncology Group (RTOG). Based on the patient's condition, either definitive doses or palliative doses were conducted. The dose constraints of lung and adjacent organs at risk (OARs) were as follows: total lungs: V5 < 60%, V20 < 30%, V30 < 20%, median lung dose (MLD) <20 Gy; heart: V30 < 40%, V40 < 30%; spinal cord <48 Gy. Vx was defined as the percentage of the total lung volume receiving no less than x Gy of radiation. Generally, the majority of patients received TRT once daily and five times per week with several SCLC patients receiving twice daily. Considering the differences in fractions, the prescribed physical dose was converted into biological effective dose (BED). The specific transformation formula is as follows: BED = D (1 + d/(α/β)), where the meaning of D = total dose, d = dose per fraction, and α/β = ratio of 10 Gy.^14^

### 2.3 Pulmonary toxicity assessment

Patients were monitored closely during TRT and routinely followed up after completion of TRT. The pulmonary toxicity mainly treatment‐related pneumonitis was assessed and diagnosed based on clinical symptoms, physical examinations, laboratory tests, and chest radiographic findings. Cumulative incidence of pneumonitis was evaluated from the date of initiation of TRT to the date of pneumonitis occurrence or the date of last follow‐up. The grade of pneumonitis was determined in accordance with the Common Terminology Criteria for Adverse Events (CTCAE), version 5.0. The record of pneumonitis grade refers to the highest grade of pneumonitis that patients suffer from. Symptomatic treatment‐related pneumonitis was grade ≥2 with new or progressive respiratory symptoms. Grade ≥3 was defined as severe pneumonitis that warranted oxygen inhalation and complicated intervention.

### 2.4 Statistical analysis

Descriptive statistics were used to show the distribution of baseline and clinical characteristics of patients with counts and percentages for categorical variables as well as median and interquartile range (IQR) for continuous variables. Univariate and multivariate logistics regression analyses were performed to identify the risk factors of grade ≥2 symptomatic pneumonitis. All continuous variables were converted to categorical variables, generally on the basis of the optimal cut‐off value calculated by receiver operating characteristics (ROC) curves. The association between dosimetric parameter variables was tested using Pearson's correlation coefficients to exclude some highly correlated variables. Variables with a *p* value ≤0.1 in the univariate analysis were then included in the further multivariate analysis with a forward‐stepwise logistic regression test. Odds ratio (OR) with 95% confidence intervals (CIs) were calculated. Two‐sided *p* < 0.05 is considered statistically significant. All statistical analyses were performed using SPSS V23.0 (IBM Corporation).

## 3 RESULTS

### 3.1 Patients and treatment characteristics

Among the 828 patients with advanced lung cancer who were treated with at least two cycles of PD‐(L)1 inhibitor, 96 patients receiving subsequent TRT were identified. Baseline characteristics are summarized in Table 1. The median age at TRT initiation was 60 (range: 33–81). A majority of patients were males (76%) and had Eastern Cooperative Oncology Group performance status (ECOG PS) of 0–1 (97.9%). The histologic types included adenocarcinoma (34.4%), squamous carcinoma (33.3%), SCLC (29.2%), and others (3.1%). At the time of irradiation, 14.6% of patients (*n* = 14) had stage III NSCLC while 85.4% (*n* = 82) had stage IV NSCLC. Twenty six patients (27.1%) had a heavy smoking history of ≥40 pack years. Baseline CT images found 22 patients (22.9%) with mild pulmonary emphysema, six patients (6.25%) with mild interstitial pneumonia and one patient (1%) with mild pulmonary fibrosis. As for immunotherapy, a large proportion of patients (62.5%) received first‐line PD‐(L)1 inhibitor treatment and 88.5% patients administered PD‐1 inhibitor treatment. The median time from the initiation of PD‐(L)1 inhibitor treatment to TRT was 4.8 months (range: 1.6–14.1 months) with most of the patients (74%) administering no less than four cycles of PD‐(L)1 inhibitor. TRT was conducted subsequently mainly for salvage therapy with target volume mostly in the lung parenchyma plus‐associated draining lymph node (DLN) (Table 1). Forty‐seven patients received palliative TRT while 49 patients received curative TRT. Most of the patients received palliative TRT to control the progression of the primary lesion or new oligometastatic lesions in the chest. Among the patients receiving radical TRT, some local advanced patients with large tumor burden first, administered PD‐(L)1 inhibitor combined with chemotherapy to reduce the tumor burden as much as possible and then received radical TRT to control the lesions, while other advanced oligometastatic patients mainly with single distant metastasis received radical TRT when the distant metastasis was well controlled almost to complete response. The median total dose was 52 Gy (IQR 50–60 Gy) and most of each dose was 1.8–2 Gy. Overall, the median dosimetric parameters were relatively low with a median total lung V20 of 15.75% (IQR 8.48%–21.64%).

**TABLE 1:** Patient baseline and treatment characteristics

Characteristics	All patients (*n* = 96)	Patients without pneumonitis (*n* = 49)	Patients with G2 pneumonitis (*n* = 28)	Patients with G3‐5 pneumonitis (*n* = 19)
Median age at TRT, years, (range)	60 (33–81)	60 (33–78)	58 (41–81)	63 (45–78)
<65	68 (70.8)	38 (77.6)	20 (71.4)	10 (52.6)
≥65	28 (29.2)	11 (22.4)	8 (28.6)	9 (47.4)
Sex (%)				
Male	73 (76.0)	36 (73.5)	22 (78.6)	15 (78.9)
Female	23 (24.0)	13 (26.5)	6 (21.4)	4 (21.1)
Histologic type (%)				
Adenocarcinoma	33 (34.4)	17 (34.7)	8 (28.6)	8 (42.1)
Squamous cell carcinoma	32 (33.3)	15 (30.6)	12 (42.9)	5 (26.3)
Small cell cancer	28 (29.2)	15 (30.6)	7 (25.0)	6 (31.6)
Others	3 (3.1)	2 (4.1)	1 (3.6)	0
Disease stage (AJCC 8th, %)				
III	14 (14.6)	6 (12.2)	7 (25.0)	1 (5.3)
IV	82 (85.4)	43 (87.8)	21 (75.0)	18 (94.7)
ECOG PS (%)				
0	17 (17.7)	10 (20.4)	6 (21.4)	1 (5.3)
1	77 (80.2)	38 (77.6)	22 (78.6)	17 (89.5)
2	2 (2.1)	1 (2.0)	0	1 (5.3)
Smoking history, pack‐year (%)				
<40	70 (72.9)	41 (83.7)	21 (75)	8 (42.1)
≥40	26 (27.1)	8 (16.3)	7 (25)	11 (57.9)
Interstitial pneumonia/fibrosis				
Yes	7 (7.3)	2 (4.1)	2 (7.1)	3 (15.8)
No	89 (92.7)	47 (95.9)	26 (92.9)	16 (84.2)
Pulmonary emphysema				
Yes	22 (22.9%)	5 (10.2)	10 (35.7)	7 (36.8)
No	74 (77.1%)	44 (89.8)	18 (64.3)	12 (63.2)
Treatment line of PD‐(L)1 inhibitor (%)				
First‐line	60 (62.5)	27 (55.1)	19 (67.9)	14 (73.7)
Second‐line	21 (21.9)	13 (26.5)	6 (21.4)	2 (10.5)
Third‐line and more	15 (15.6)	9 (18.4)	3 (10.7)	3 (15.8)
PD‐(L)1 inhibitor type (%)				
PD‐1 inhibitor	85 (88.5)	44 (89.8)	24 (85.7)	17 (89.5)
PD‐(L)1 inhibitor	11 (11.5)	5 (10.2)	4 (14.3)	2 (10.5)
Median time from the initiation of PD‐(L)1 inhibitor treatment to TRT, month (range)	4.8 (1.6–14.1)	4.8 (1.6–14.1)	3.8 (2.0–11)	4.3 (2.1–6.3)
Cycles of PD‐(L)1 inhibitor before TRT (%)				
<4	25 (26.0)	12 (24.5)	9 (32.1)	4 (21.1)
≥4	71 (74.0)	37 (75.5)	19 (67.9)	15 (78.9)
Total dose, Gy, median (IQR)	52 (50–60)	51 (50–59.4)	53 (50–60)	52 (46–60)
BED10, Gy	64.8 (60–72)	66.3 (60–72)	64.7 (59.1–72)	64.8 (55.2–72)
GTV, cc	63.8 (25.08–139.98)	47.1 (20.5–119)	64.9 (27.5–143.1)	102.3 (35.4–165.8)
PTV, cc	233.5 (114.08–380.23)	192.9 (74.5–367.4)	296.7 (134.5–376)	257.2 (165.5–451.1)
PTV/LV	0.08 (0.04–0.13)	0.07 (0.03–0.12)	0.08 (0.05–0.13)	0.09 (0.05–0.16)
Total lung V5, %	33.95 (24.82–44.29)	27.7 (19.5–41.3)	35.6 (27.7–46)	41.6 (29.9–52.7)
Total lung V20, %	15.75 (8.48–21.64)	12.1 (5.7–19.9)	17 (12.6–21.6)	20.9 (12–23.6)
MLD, Gy	8.94 (5.84–10.66)	7.5 (4.0–9.8)	9.4 (8.1–11.2)	9.5 (7.5–11.3)
TRT Target area (%)				
Lung Parenchyma	30 (31.3)	21 (42.9)	5 (17.9)	4 (21.1)
Thoracic Lymph Nodes	10 (10.4)	5 (10.2)	3 (10.7)	2 (10.5)
Lung parenchyma and Nodes	56 (58.3)	23 (46.9)	20 (71.4)	13 (68.4)

Abbreviations: ECOG PS, Eastern Cooperative Oncology Group performance status; PD‐(L)1, programmed death 1/programmed death ligand 1; TRT, thoracic radiotherapy; IQR, interquartile range; BED10 biologically effective doses; GTV gross tumor volume; PTV planning target volume; LV total lungs volume; Vx: percentage of total lungs volume receiving no less than x Gy; MLD: mean dose of total lungs.

Of the six patients suffering from fatal pneumonitis, their detail baseline characteristics are depicted in Table 2. All patients were males, had a ECOG PS of 0–1 and had a smoking history with four patients ≧40 pack years. Five of the six patients received immunotherapy as first‐line treatment. Besides, all patients administered no less than 4 cycles of PD‐1 inhibitor treatment. The total doses of radiation varied (39–60 Gy) with mainly targeting parenchyma plus draining lymph node.

**TABLE 2:** Baseline and treatment characteristics of patients with fatal pneumonitis

Characteristics	Patient 1	Patient 2	Patient 3	Patient 4	Patient 5	Patient 6
Patient baseline characteristics						
Age (years)	55	66	70	78	62	68
Sex	M	M	M	M	M	M
Histologic type	Small cell	Adenocarcinoma	Squamous	Adenocarcinoma	Squamous	Squamous
Disease stage	Ⅳ	Ⅳ	Ⅲ	Ⅳ	Ⅳ	Ⅳ
ECOG PS	0	1	1	1	1	1
Smoking history (Pack‐years)	1	125	20	50	40	75
Pulmonary emphysema	N	Y	N	N	N	Y
Interstitial pneumonia/fibrosis	N	Y	N	Y	N	N
Treatment line of PD‐(L)1 inhibitor	1	1	1	1	2	1
PD‐(L)1 inhibitor type	PD‐1	PD‐1	PD‐1	PD‐1	PD‐1	PD‐1
Cycles of PD‐(L)1 inhibitor before TRT	4	11	6	5	6	9
Time from the initiation of PD‐(L)1 inhibitor treatment to TRT (months)	6.0	9.5	4.3	3.2	3.7	6.8
Dosimetric parameters of TRT						
Total dose/fractions	50 Gy/25f	60 Gy/30f	60 Gy/30f	55.8 Gy/31f	52 Gy/26f	36 Gy/20f
Total lung V5 (%)	36.05	33.43	56.31	40.05	52.71	44.48
Total lung V20 (%)	23.17	15.71	25.37	12	21.57	18.02
MLD (Gy)	11.28	8.82	13.05	8.26	11.43	9.08
TRT Target area	Parenchyma+DLN	Parenchyma+DLN	Parenchyma	Parenchyma+DLN	Parenchyma+DLN	Parenchyma+DLN

Abbreviations: M, male; F, female; Y, yes; N, no; ECOG PS, Eastern Cooperative Oncology Group performance status; PD‐(L)1, programmed death 1/programmed death ligand 1; TRT, thoracic radiotherapy; Vx, percentage of total lungs volume receiving no less than xGy; MLD, mean dose of total lungs; DLN, draining lymph node.

### 3.2 Occurrence and characteristics of treatment‐related pneumonitis

**FIGURE 1:**
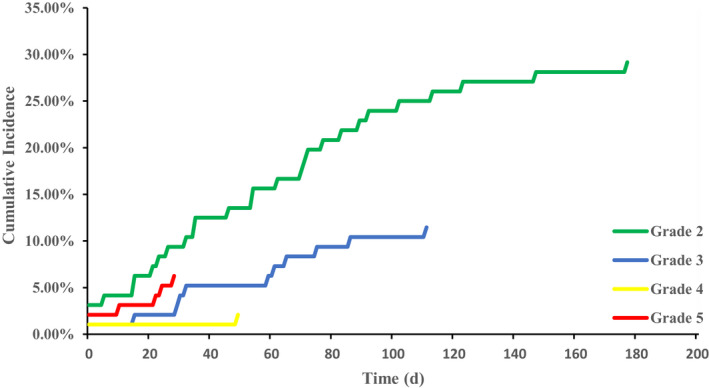
Cumulative incidence of treatment‐related pneumonitis by grade

Median follow‐up time from the completion of TRT was 8.6 months (range: 4.5–20 months). During follow‐up, 47 patients (48.96%) developed symptomatic treatment‐related pneumonitis (grade ≥2). The incidence rate of grade ≥3 severe pneumonitis was 19.79% with six patients (6.25%) suffering from fatal toxicities. The median time of pneumonitis onset after completion of TRT was 35 days (range 0–177 days) with six patients developing during TRT. Notably, all fatal pneumonitis occurred within 1 month after TRT. All of the six patients who developed pneumonitis during TRT discontinued TRT immediately at the onset of pneumonitis symptoms. Figure 1 showed the cumulative incidence of pneumonitis by grade.

**FIGURE 2:**
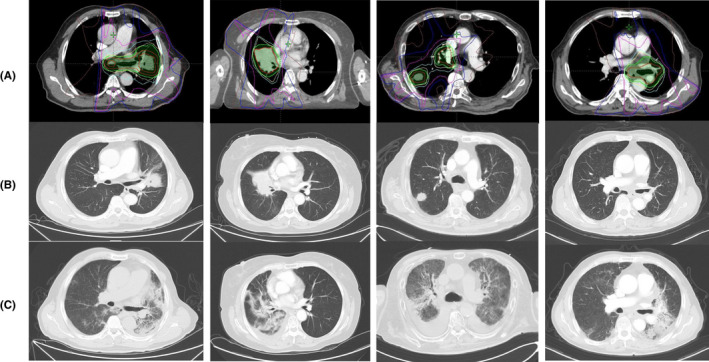
Representative CT images of four patients with symptomatic pneumonitis. (A) Radiation planning imaging with target volume and dose distribution of TRT (B) CT images before TRT; (C) CT images at time of diagnosis of symptomatic pneumonitis

The symptomatic pneumonitis mainly manifested as nonproductive cough (*n* = 40), chest distress (*n* = 28), and dyspnea (*n* = 24). Several patients also presented with hypoxia (*n* = 14), fever (*n* = 9), and chest pain (*n* = 5). Radiographic patterns of the pneumonitis were varied mainly including ground‐glass opacities (*n* = 20), patchy consolidation (*n* = 16), and other interstitial changes (*n* = 8). Notably, although most of the patients had pneumonitis mainly located in high RT dose regions, there were several patients whose pneumonitis was observed within low RT dose regions or outside the dose fall‐off regions. Representative chest CT images are illustrated in Figure 2. All of the 47 patients with symptomatic pneumonitis receiving corticosteroid management with a median dose of 80mg (range 40–160 mg).

### 3.3 Potential risk factors for treatment‐related pneumonitis

Multiple variables including patient‐related, tumor‐related, and dosimetric factors were included in the univariate analysis. The results of the univariate analysis are shown in Table 3. Totally, smoking history, pulmonary emphysema, planning target volume (PTV), planning target volume/lung volume (PTV/LV), V5, V20, and MLD were significantly associated with symptomatic pneumonitis. The optimal cut‐off values of PTV, PTV/LV, V5, V20, and MLD to predict the occurrence of pneumonitis were 223 cm^3^, 0.08, 32.3%, 16.7%, and 9.1 Gy respectively, as determined by ROC curves.

**TABLE 3:** Univariate analysis of risk factors for symptomatic pneumonitis

Factors	Univariate analysis	
	OR (95% CI)	*p* value
Sex		
Male	0.75 (0.29–1.92)	0.547
Female		
Age		
<65	1.96 (0.80–4.80)	0.142
≥65		
Histologic type		0.861
Adeno (ref.)		
Squamous	1.06 (0.41–2.77)	0.901
Small cell	0.81 (0.30–2.19)	0.682
Disease stage		
III	0.68 (0.22–2.14)	0.509
IV		
ECOG PS		
0	1.54 (0.53–4.48)	0.423
1		
Smoking history		
<40	3.18 (1.22–8.30)	0.018
≥40		
Pulmonary emphysema		
Yes	4.99 (1.66–14.98)	0.004
No		
Interstitial pneumonia/fibrosis		
Yes	2.8 (0.52–15.19)	0.233
No		
Treatment line of PD‐(L)1 inhibitor		
1	0.52 (0.22–1.21)	0.128
≥2		
PD‐(L)1 inhibitor type		
PD‐1 inhibitor	1.29 (0.36–4.54)	0.694
PD‐L1 inhibitor		
Cycles of PD‐(L)1 inhibitor before TRT (%)		
<4	0.85 (0.34–2.11)	0.724
≥4		
BED10 (Gy)		
<62.45	0.61 (0.27–1.37)	0.228
≥62.45		
Time from the initiation of PD‐(L)1 inhibitor treatment to TRT (months)		
<4.8	0.49 (0.22–1.13)	0.095
≥4.8		
GTV (cm^3^)		
<58	1.84 (0.81–4.22)	0.148
≥58		
PTV (cm^3^)		
<223	2.42 (1.04–5.59)	0.039
≥223		
PTV/LV		
<0.08	2.65 (1.14–6.15)	0.024
≥0.08		
Total lung V5 (%)		
<32.3	3.2 (1.36–7.53)	0.008
≥32.3		
Total lung V20 (%)		
<16.7	3.57 (1.51–8.45)	0.004
≥16.7		
MLD (Gy)		
<9.1	2.48 (1.06–5.77)	0.036
≥9.1		

Abbreviations: BED10, biologically effective doses; CIs, confidence intervals; ECOG PS, Eastern Cooperative Oncology Group performance status; GTV, gross tumor volume; LV, total lungs volume; MLD, median lung dose; OR, Odds ratio; PD‐(L)1, programmed death 1/programmed death ligand 1; PTV, planning target volume; ref, reference; TRT, thoracic radiotherapy; Vx, percentage of the total lung volume receiving no less than x Gy of radiation.

Factors with a *p* value no more than 0.1 on univariate analysis were then considered to be included in further multivariate analysis. Considering that all dosimetric parameters might be correlated with each other, Pearson's correlation coefficient was calculated to confirm the relationship between the parameters. Collectively, V5, V20, and MLD were highly correlated with each other while PTV and PTV/TLV also showed strong correlation. The Pearson's correlation coefficients between V20/V5, V20/MLD, and V5/MLD were 0.854, 0.957, and 0.893, respectively, whereas the correlation coefficient between PTV and PTV/LV was 0.871. Based on the clinical significance and statistical *p* values, V20 and PTV/LV was selected as representatives of dosimetric parameters for further multivariate analysis. Ultimately, variables comprising smoking history, pulmonary emphysema, time from the initiation of PD‐(L)1 inhibitor treatment to TRT, total lung V20, and PTV/LV were tested in multivariate analysis. The result revealed that pulmonary emphysema and V20 were independent risk factors of symptomatic pneumonitis (OR: 5.67, 95% CI: 1.66–19.37, *p* = 0.006; OR: 3.49, 95% CI: 1.41–8.66, *p* = 0.007, respectively) (Table 4). The cumulative incidence of symptomatic pneumonitis was 77.27% in patients with pulmonary emphysema while 40.54% in patients without pulmonary emphysema. And for V20, the cumulative incidence of symptomatic pneumonitis was significantly higher in patients with V20 ≥16.7% compared with those <16.7%, (67.44% vs. 36.73%).

**TABLE 4:** Multivariate analysis of risk factors for symptomatic pneumonitis (By forward‐stepwise regression test)

Factors	OR (95% CIs)	*p* value	Beta coefficient
Pulmonary emphysema	5.67 (1.66–19.37)	0.006	1.735
V20 (<16.7%/≥16.7%)	3.49 (1.41–8.66)	0.007	1.250
Smoking history (<40/≥40 pack years)	–	0.376	–
PTV/LV (<0.08/≥0.08)	–	0.308	–
Time from the initiation of PD‐(L)1 inhibitor treatment to TRT (<4.8/≥4.8 months)	–	0.076	–

Abbreviations: CIs, confidence intervals; LV, total lungs volume; OR, Odds ratio; PD‐(L)1, programmed death 1/programmed death ligand 1; PTV, planning target volume; TRT, thoracic radiotherapy; Vx, percentage of the total lung volume receiving no less than x Gy of radiation.

## 4 DISCUSSION

To the best of our knowledge, this is the first study to report and characterize the safety of TRT subsequently after multicycles of PD‐(L)1 inhibitor treatment in patients with lung cancer. We observed a substantially increased incidence and severity of treatment‐related pneumonitis (grade ≥2, 48.96%; grade ≥3, 19.79%) after completion of TRT with 6.25% patients developing fatal pneumonitis. Additionally, we identified that pulmonary emphysema and total lung V20 were independent risk factors associated with the occurrence of symptomatic pneumonitis.

Radiation pneumonitis is one of the most severe toxicities of TRT. It has been reported that the risk of ≥grade 2 symptomatic pneumonitis range from 5% to 30% with TRT alone or combined with platinum‐based chemotherapy.^7^, ^15^, ^16^, ^17^ PD‐(L)1 inhibitors, which reinvigorate anti‐tumor immune response, could also cause damage to normal lung tissue with reported occurrence of immune‐related pneumonitis from 3% to 5%.^18^, ^19^, ^20^ Theoretically, combination regimen of PD‐(L)1 inhibitor and TRT irrespective of concurrent or sequential could induce overlapped pulmonary toxicities, possibly presenting with appreciably increased incidence of pneumonitis. Previous prospective and retrospective studies revealed that the occurrence of all grade treatment‐related pneumonitis varied from 8.2% to 33.9% with grade≥3 pneumonitis ranging from 3.4% to 11.1% in patients with lung cancer receiving PD‐(L)1 inhibitor treatment concurrently combined with TRT or sequentially with a history of TRT, which was considered tolerable overall.^2^, ^9^, ^21^, ^22^, ^23^ By contrast, in our study, we found that TRT sequentially after multicycles of PD‐(L)1 inhibitor treatment induced a remarkably higher incidence and severity of pneumonitis, which required more clinical attention and vigilance.

The increasing use of PD‐(L)1 inhibitor particularly in first‐line treatment in patients with advanced lung cancer potentially leads to a situation, in which definitive or palliative doses of TRT are conducted in close sequential succession for consolidation therapy or salvage therapy. Nonetheless, there is a paucity of literature related to this sequential regimen. Yuan Z. et al. presented a case that palliative TRT‐induced potent antitumor immune response in a patient who was nivolumab refractory, which first, demonstrated the viability of the sequential regimen.^24^ No adverse event was reported in this case. A retrospective study by N. Shaverdian et al. assessed the safety of TRT in patients with prior irAEs from previous ICIs.^25^ Forty‐one patients with various tumor types including NSCLC, melanoma, sarcoma, ovarian, and others were selected for toxicity assessment. The results indicated that patients with prior irAEs might be at high risk for developing clinically significant and persistent ≥grade 2 pneumonitis from TRT (25 of 41, 61%). Our study focused on lung cancer and included all patients with previous PD‐(L)1 inhibitor treatment who underwent subsequent TRT regardless of with prior irAEs history or not. We observed a substantially increased incidence of pneumonitis (47 of 96, 48.96%) as well and of particular concern, a relatively high incidence of fatal pneumonitis (6 of 96, 6.25%), which reminded oncologists and researchers to raise great awareness of potentially increased pulmonary toxicity from this combination regimen in patients with lung cancer.

The potential mechanisms of the substantially increased incidence of pneumonitis induced by this sequential regimen are complicated and unclear. Generally, the development of treatment‐related pneumonitis is mainly attributed to uncontrolled pro‐inflammatory responses including overwhelming release of cytokines and reactive oxygen species (ROS).^26^, ^27^ Prior PD‐(L)1 inhibitor treatment could reprogram the tumor microenvironment by normalizing tumor vasculature, improving tissue perfusion and decreasing intratumoral hypoxia in a T cell‐dependent manner, which may potentially sensitize the tumor to TRT and meanwhile makes surrounding healthy lung tissue more fragile to attack of TRT.^13^, ^28^, ^29^, ^30^ Further research requires a more in‐depth understanding of the complex interactions of PD‐(L)1 inhibitor and TRT in tumor microenvironment and surrounding healthy tissue.

In our study, we found that pulmonary emphysema on baseline CT images was significantly associated with symptomatic pneumonitis. The incidence of symptomatic pneumonitis was substantially higher in patients with pulmonary emphysema than those without (77.27% vs. 40.54%). Previous studies have reported that pulmonary emphysema was an independent risk factor for symptomatic pneumonitis after TRT.^31^, ^32^ For instance, Zhou Z. et al. retrospectively analyzed 153 patients with locally advanced NSCLC who received definitive TRT.^31^ The results showed that patients with pulmonary emphysema had an increased risk for developing radiation pneumonitis regardless of grade ≥2 (OR = 1.985, *p* = 0.01) or grade ≥3 (OR = 2.275, *p* = 0.02). Generally, patients with pulmonary emphysema or chronic obstructive pulmonary disease were prone to have relatively impaired pulmonary function which might make patients more susceptible to developing persistent even severe pneumonitis after PD‐(L)1 inhibitor treatment or TRT.^33^ Therefore, caution must be emphasized on potentially undue pulmonary toxicities for patients with pulmonary emphysema when receiving TRT following prior multicycles of PD‐(L)1 inhibitor treatment. Notably, of the seven patients with preexisting interstitial pneumonia/fibrosis, five (71.4%) developed symptomatic pneumonitis. Patients with preexisting interstitial pneumonia/fibrosis may be more prone to symptomatic pneumonitis when receiving the combination treatment. Considering the small sample size of patients with preexisting interstitial pneumonia/fibrosis in our study, this risk factor was not demonstrated to be correlated with symptomatic pneumonitis. However, our further study is ongoing to explore the effect of preexisting mild interstitial pneumonia/fibrosis on the occurrence of symptomatic pneumonitis especially severe pneumonitis in lung cancer patients who received this sequential regimen.

The total lung V20 is a crucial and the most frequently described dosimetric parameter generally used to define a specific threshold dose to predict the occurrence of radiation pneumonitis.^15^, ^16^, ^34^, ^35^, ^36^, ^37^, ^38^ For example, Palma et al. performed a meta‐analysis of 836 patients with lung cancer who underwent concurrent chemoradiotherapy and evaluated the association between V20 and symptomatic pneumonitis.^15^ The results showed that V20 = 25% was an optimal cut‐off value to differentiate patients with high risk for development of symptomatic pneumonitis (OR 1.03, *p* = 0.008). A multicenter real‐world study conducted by Takashi et al. revealed that lung V20 ≥ 26% was correlated with high incidence of symptomatic pneumonitis in NSCLC patients treated with concurrent chemoradiotherapy and subsequent consolidation durvalumab immunotherapy. The 12‐month incidence of symptomatic pneumonitis was up to 50% with lung V20 ≥ 26%, compared to 27% with lung V20 < 26%.^34^ Consistently, our results also indicated that V20 was an independent risk factor for occurrence of symptomatic pneumonitis (OR 3.49 *p* = 0.007; V20 ≥ 16.7% vs. V20 < 16.7%, 67.44% vs. 36.73%). Notably, the cut‐off value of V20 in our analysis was lower than those established in previous studies with range of 20%–35%.^15^, ^16^, ^34^, ^35^, ^36^, ^37^, ^38^ We speculated that this was possibly due to the predisposition of symptomatic pneumonitis in patients receiving TRT after PD‐(L)1 inhibitor treatment, which alerted radiation oncologists to delineate target plan carefully to control total lung V20 in order to minimize the incidence of pneumonitis when chose this sequential regimen.

This study has several limitations. First, it was a retrospective study from a single medical center. Inevitably, there existed potential bias. So the conclusion should be interpreted with caution. Nonetheless, this study was of great significance to alert oncologists to be cautious when chose this sequential regimen. Second, patients in our study received PD‐(L)1 inhibitor treatment combined with subsequent TRT, given that the specific standards to diagnose immune‐related pneumonitis or radiation pneumonitis were uncertain, so we did not differentiate the treatment‐related pneumonitis specifically. However, whether it is immune‐related pneumonitis or radiation pneumonitis or both, corticosteroid therapy should be given once symptoms developed. Third, most patients in our study had not yet achieved a survival outcome, so there is a lack of finding of the impact of pneumonitis induced by this sequential regimen on patient survival. But further exploration is ongoing, which worth expecting.

In conclusion, we revealed that TRT after multicycles of PD‐(L)1 inhibitor treatment induced a significantly increased incidence and severity of treatment‐related pneumonitis in patients with lung cancer. Preexisting pulmonary emphysema and total lung V20 were confirmed to be independent risk factors for development of symptomatic pneumonitis in our study. It matters to weigh the risks against the benefits of TRT in patients with prior PD‐(L)1 inhibitor treatment. Close monitoring, prompt diagnosis, and early intervention should be emphasized to mitigate the undue pulmonary toxicity if choose this sequential combination. Furthermore, large prospective clinical trials with longer and rigorous follow‐up are warranted.

## ETHICAL APPROVAL CONSENT TO PARTICIPATE

This study was approved by the IRB of Shandong Cancer Hospital and Institute. Due to the retrospective design, no separate informed consent was obtained in this study.

## CONSENT FOR PUBLICATION

All authors have consent for publication of this study.

## CONFLICT OF INTEREST

The authors declare that they have no competing interests.

## AUTHORS’ CONTRIBUTIONS

Xiangjiao Meng and Jinming Yu: Conceptualization, Supervision, and Writing—Review & Editing. Yu Chen: Data curation, Formal analysis, and Writing—Original draft preparation. Zhaoqin Huang, Kaikai Zhao, and Yao Wang: Visualization and Investigation. Xinchao Liu and Fei Ren: Investigation and Resources.

## Data Availability

The corresponding authors had full access to all the data in the study. Individual participant data will not be available unless reasonable request to the corresponding authors.
